# Organizational Solutions to the Moral Risks of Policing

**DOI:** 10.3390/ijerph17207461

**Published:** 2020-10-14

**Authors:** Daniel M. Blumberg, Konstantinos Papazoglou, Michael D. Schlosser

**Affiliations:** 1 California School of Professional Psychology, Alliant International University, San Diego, CA 92129, USA; 2 Department of Criminal Justice, New Jersey City University, Jersey City, NJ 07305, USA; kpapazoglou@njcu.edu; 3 Police Training Institute, University of Illinois at Urbana-Champaign, Champaign, IL 61820, USA; schlossr@illinois.edu

**Keywords:** police wellness, moral risks, police misconduct, police leadership

## Abstract

In addition to the physical and emotional challenges faced by law enforcement professionals, the job confronts officers with numerous moral risks. The moral risks include moral distress, moral injury, ethical exhaustion, compassion fatigue, and practices that lead to lapses in ethical decision-making. The paper focuses on what police agencies can do to better address the moral risks of policing. These moral risks are central to officer wellness and, thus, a crucial component of officers’ operational readiness. Strategies are presented that will improve prevention efforts, including recruiting and hiring, training, supervision, and promotional practices. Additionally, the paper offers recommendations for effective approaches to intervention with officers who have displayed the effects of these moral risks. Finally, the paper highlights the kind of law enforcement leaders who are best able to implement strategies designed to prevent negative outcomes associated with the moral risks of policing.

## 1. Introduction and Statement of the Problem

There are well-understood, but under-researched moral risks inherent in routine police work [1,2,3]. These risks take two paths, which synergistically impact each other. The first path contains the moral risks that increase the likelihood that officers will experience emotional and spiritual difficulties. For example, moral distress, a phenomenon that has received considerable empirical attention in the nursing and healthcare fields [4,5], results from a sense of powerlessness that police officers experience when they are unable to sufficiently help everyone seeking police assistance. Moral distress also occurs when officers experience a conflict between what they believe is morally right and what they are ordered by their supervisors to do or by what the organization’s policies mandate them to do. Similarly, many officers experience ethical exhaustion. Ethical exhaustion, or compassion fatigue, has been described as “the cost of caring” ([6], p. 9), which leaves officers feeling helpless and powerless to alleviate others’ suffering. This is differentiated from emotional exhaustion [7,8,9], whereby the emotional labor exerted to display emotions to the public other than those that an officer actual feels leads to exhaustion and burnout.

The second path contains the moral risks that increase the likelihood that officers will engage in misconduct. Numerous theories about unethical decision-making have been studied in fields other than law enforcement, with less empirical attention of these theories applied to police officers. However, each of the theories finds support in the routine operational conduct of police officers [2]. These moral risks include moral disengagement [10], moral compromise [11], moral licensing [12], and the slippery slope [13].

The two paths converge and impact each other. Some of the moral risks that cause officers to experience emotional and/or spiritual distress lead to lapses in ethical decision-making. Likewise, officers’ emotional and spiritual distress is exacerbated by their acts of misconduct. For example, research has demonstrated that emotional exhaustion can lead to increased incidence rates of depression and anxiety among police officers [14]. Other research, not on a police sample, showed a relationship between anxiety and increases in unethical behavior [15]. Thus, although not yet empirically validated, police executives should be concerned that officers who experience work-related anxiety may be more prone to engage in unethical behavior.

Similarly, Papazoglou et al. [16] showed a relationship between moral injury [17] and incidence rates of post-traumatic stress disorder (PTSD). That connection can occur when the moral injury resulted from an incident or incidents in which the officer was exposed in some way to death, threatened death, actual or threatened serious injury, or actual or threatened sexual violence [18]. Moreover, beyond how moral injury is commonly conceptualized, there are additional ways in which it occurs in policing. Police officers can experience a moral injury when they are ordered to perform enforcement actions that run contrary to their personal values, which leaves them with feelings of guilt or shame. It can also result from feelings of anger when officers feel betrayed by the behavior of trusted colleagues and/or supervisors, including when supervisors give orders that officers view as morally wrong. Moreover, moral injury can result from the behavior of officers whenever they do something (or fail to do something) that violates their core values, which is independent of the orders of a supervisor. This behavior can be intentional without thinking about the subsequent reactions that the officer might experience. The behavior also can be unavoidable due to circumstances beyond the officer’s control (e.g., not being able to get to a call in time to stop its tragic outcome). Moreover, the behavior that leads to a moral injury can occur because of an officer’s error or momentary lapse in judgment.

Thus, moral distress, compassion fatigue, ethical exhaustion, and PTSD can result from and lead to officers relaxing their commitment to ethical principles. Specifically, when officers’ commitment to ethical principles is compromised, they are more prone to engage in behavior that leads to a moral injury. The moral injury leaves officers overwhelmed with guilt, shame, and/or anger with potentially harmful results. It remains to be empirically investigated, but it is clinically reasonable to conclude that police officers suffering from the intense guilt and shame from a moral injury are more at risk for suicidal ideation and behavior.

Although mental health providers who work with law enforcement personnel have begun to pay more attention to the moral suffering of their police patients, there remains a lack of attention to the moral risks of policing from police executives. Most agencies spend a lot of time and energy adjudicating officer misconduct, but few recognize that much of this unethical behavior is preventable. In fact, some of the misconduct is fostered by the agency’s culture and by actions of the agency’s supervisors. Other unethical behavior is a natural by-product of routine policing, such as the use of discretion without impartiality. Similarly, few law enforcement executives have taken steps to address and to mitigate their officers’ moral suffering, when much of this distress stems from officers’ chronic exposure to critical and traumatic incidents.

Law enforcement organizations can change the culture to better address the various moral risks faced by their officers. This starts at the top. Police leadership must commit to tackling this complex and multifaceted problem. The comprehensive approach to culture change is two-pronged, focusing on prevention and intervention strategies. Police executives need to recognize the deleterious consequences of the moral risks of policing on their officers’ overall health and wellness. It is not enough to focus on officer wellness without concurrently attending to these moral risks and the ensuing damage they inflict.

Specific prevention strategies will be offered. The paper discusses steps that agencies can take to recruit and to hire officers who are more likely to maintain their commitment to ethical principles. However, given current hiring challenges, attention must be paid to training efforts that are necessary to boost recruits’ resilience and to strengthen their ability to successfully navigate the often choppy ethical waters of policing. For example, current innovative training practices that focus on strengthening recruits’ ethical decision-making [19] and emotional intelligence [20] are presented. Additionally, the need for agencies to install dedicated wellness programs with adequately resourced wellness officers [21] is discussed. A strong case is made that these wellness programs must include a focus on the moral risks of policing [3,22].

Additionally, intervention strategies are offered. Beyond the officer wellness efforts mentioned above, this focuses on ways in which agencies should implement early detection methods. Early detection centers on the two paths of moral risks. Peer support, proactive wellness efforts, availability of mental health services, and supervision practices that attend to the moral risks of policing are meant to intervene at the first signs of officers’ emotional and/or spiritual distress. Additionally, the argument is made that promotional practices, including selection of training officers, must include attention to a candidate’s willingness and ability to confront indications of their officers’ distress in a manner consistent with the agency’s culture of wellness and ethics [23].

At the same time, intervention must address the risks that increase the likelihood of officer misconduct. The paper presents ways in which agencies can revamp oversight and discipline [2,24,25] in an effort to intervene at the first indication of officers’ ethical lapses. Recognizing the obstacles that this approach faces vis-à-vis police unions and collective bargaining agreements (CBAs), some solutions are offered. These will include: engaging union officials; negotiating certain “improvements” in future CBAs, which center on the moral risks of policing; and, expanding the focus beyond the organization, to include members of the community.

## 2. Prevention Efforts

When law enforcement leaders recognize the various ways in which the moral risks of policing impact their officers, greater attention can be paid to prevention efforts. These efforts simultaneously focus on minimizing officers’ emotional and spiritual distress and on deterring officers’ unethical behavior. It is crucial to integrate these strategies and to see them as a coordinated attempt to mitigate the negative impact of the moral risks. However, for clarity, the suggestions discussed here are presented separately.

### 2.1. Recruiting and Hiring

Unlike decades past when law enforcement agencies had the luxury to hire a select few candidates from a large pool of qualified applicants, the sad fact is that many agencies these days have been forced to lower their hiring standards because of a shrinking pool of applicants. Euphemistically, police leaders will say that they have broadened hiring criteria. The result is that many agencies, especially large ones, are now resigned to hiring the cream of the crop from the bottom of the barrel. This poses a myriad of challenges once new hires are onboarded. One of these challenges, particularly when it comes to new hires who have minimal life experience, is a greater vulnerability to experience the negative effects of the moral risks of policing. This makes it especially important for agencies to consider ways to avoid negative outcomes of the moral risks in relation to recruiting and hiring of new officers.

#### 2.1.1. Minimizing Emotional and Spiritual Distress

Although most veteran officers are susceptible to these moral risks, some new hires will be particularly prone to develop emotional and/or spiritual distress from the moral risks of policing. Many of these new hires are the “green” recruits who were hired without much prior life experience. They have limited independent living experience, limited job experience, and limited, if any, related work, educational, or volunteer experience. They were hired because there was no disqualifying evidence found in their background investigation. They gave a confident interview to the hiring authority. Moreover, there was nothing disqualifying that surfaced during their pre-employment psychological examination. Additionally, other new recruits who are vulnerable to the moral risks seem, during the hiring process, to have adequate prior experience and preparation for a law enforcement job. Some have worked in security. Some have degrees in administration of justice. Some were in the military. Nevertheless, both groups of these new hires often have unrealistic expectations about what the job entails. They are ill-prepared for the constant exposure to the pain and suffering of others. They have never been required to follow procedures and orders that violate their personal values. They have never worked in a position with the level of ambiguity and multitasking required of police officers. Unfortunately, they are in for a very rude awakening when their expectations are fairly quickly shown to be overly idealistic.

There are not many things that can adequately prepare someone for a career in law enforcement. However, this should not stop police leaders from implementing improvements during the recruiting and hiring phases that will help to minimize the impact of the moral risks on their future employees. The first thing is to “Shift the narrative to show the realities of the profession and provide clear expectations to potential applicants” ([26], p. 2). This is a tough balancing act when attempting to attract people to the profession, but a necessary step to take. Your recruiters and background investigators should not be salespersons who romanticize the job. They should not be incentivized by the number of new hires they bring to the agency. Moreover, your efforts should go beyond a realistic narrative.

Applicants should be exposed to the various rigors and mundanities of the job. Rather than rushing through the hiring process, police leaders will be better served by slowing down and providing applicants with a clearer, more realistic understanding of what lies ahead. Some of this should be early in the process to prevent unnecessary expenditure of resources. For example, agencies can create a series of recruiting videos. The first one casts the widest net and goes on YouTube and runs as an ad spot during the local television news broadcasts; it shows all the toys and makes the job seem fun and exciting. The second one is required viewing for those who submit an application and explains some of the less fun and less exciting aspects of the job, while still highlighting many of the benefits of working for the agency. If you lose some applicants after they see that video, you saved yourself a lot of time and money. Then, the third video is shown to applicants who pass the hiring steps prior to opening the background investigation. This video can be a heartfelt, personal message from the Chief, which thanks them for their interest, gives them several reasons why they will love working there, and encourages them to think about why this might not be the job for them.

Once applicants enter the background investigation phase of the hiring process, agencies can continue the pre-hire socialization process by requiring applicants to complete two or three ride-alongs. This is unparalleled exposure to the reality of police work. Applicants’ background investigators should conduct a debriefing after each ride-along and ask a standard set of questions, such as the following:

- What did you see on the ride-along that surprised you?
- Which calls for service got you excited to be an officer?
- Which calls for service seemed like they might be most difficult for you?
- Which calls for service had what you would consider a negative outcome?

The ride-alongs will ideally expose applicants to both busy and quiet shifts, giving a realistic picture of the job at your agency. During this phase of the hiring process, some agencies invite applicants to attend organized pre-academy workouts. During these activities, the staff who supervise the workouts should encourage discussion among the applicants of their ride-along experiences. Rather than running these workouts in a para-military fashion (with the intention of getting them ready for what they will encounter in the academy), it would be more beneficial to use the workouts as an additional opportunity to present and discuss the ups and downs of the job.

Having provided applicants with realistic expectations, after giving a conditional offer of employment, agencies can consider implementing a pre-academy experience. The goal now is not to weed out those applicants who are likely to quit when their expectations are not met. Instead, the goal is to increase retention of these applicants to whom you have dedicated so much time and resources during the hiring process. The pre-academy is designed to boost applicants’ resilience and strengthen their resolve to remain in the profession despite the emotional and spiritual challenges that they will encounter. This can be a two-day to five-day experiential pre-academy in which the soon-to-be recruits are trained to use a variety of resilience and personal strengths building exercises (see [27]). Veteran and retired officers facilitate the pre-academy and demonstrate ways in which they coped with the emotional and spiritual risks they faced. This also is where psychological services that are available to agency employees and the professionals who provide them can be introduced. Additionally, the pre-academy is where agencies can obtain support from the new hires’ family members, who often add additional stress to recruits because of their fear for their loved one’s safety. The pre-academy can schedule a time for family members to participate, to learn about resources available to them, to hear police-family success stories, and to learn about some of the early warning signs of distress that their loved one may exhibit [28]. Enlisting the participation of family members is an important retention tool.

#### 2.1.2. Deterring Misconduct

The moral risks that increase the likelihood that officers will engage in misconduct should begin to be addressed during the recruiting and hiring process. The fundamental objective is to recruit and to hire applicants with a proven track record of ethical behavior. It is especially relevant to see that applicants did the right thing even when it was not easy, when there were competing temptations, and when they faced pressure or ridicule from peers. Unfortunately, many applicants have not displayed such resolute character traits. In fact, agencies often hire applicants with a history of an “acceptable” number and/or type of delinquent behavior in their pasts. The common refrains are that such behavior was “youthful indiscretion,” or “all college students do that,” or “that was just boys being boys.” In some cases, the hiring authority discounts delinquent acts when the applicants subsequently served a military enlistment without any disciplinary actions. However, it is unknown whether or not the applicant matured during the military; conversely, it is possible that the structure of the military kept them on the straight and narrow while serving, but they could stray once discharged and back in their neighborhood with the old distractions. Instead, it is essential to see evidence of applicants’ more recent pattern of ethical behavior under circumstances that are similar to when the earlier unethical or illegal behavior occurred [29]. Lastly, it is unwise to hire applicants who are a “blank slate” when it comes to ethical behavior. If they have not been ethically or morally tested (i.e., they have not had an opportunity to prove that they will do the right thing no matter what), then the agency runs the risk of hiring someone who will be very vulnerable to the moral risks of policing.

The key characteristic to look for when recruiting and hiring with an eye on the moral risks is integrity. Philosophy students can argue about what it means to live with integrity. When it comes to hiring an ethical applicant, this should not be open to debate. California POST [30] defines integrity as follows:

*maintaining high standards of personal conduct. It consists of attributes such as honesty, impartiality, trustworthiness, and abiding laws, regulations, and procedures. It includes: not abusing the system or using one’s position for personal gain; not bending rules or otherwise trying to beat the system; and, not engaging in illegal or immoral activities either on or off the job.*

(p. 61)

Unfortunately, when background investigators use this definition, it provides little guidance about what to look for in an applicant. It is clear about what the applicant should not have done, which indicates a lack of integrity, but it does not give examples of how a background investigator can assess honesty, impartiality, and trustworthiness. Therefore, agencies check this box by setting their sometimes arbitrary parameters for applicants’ past illegal or unethical behavior, such as the number of moving traffic citations, use of illegal drugs, and incidence of shoplifting or cheating in school. These parameters typically involve both the number of past incidents as well as the recency of them prior to applying to the job. Then, a hiring decision is made in conjunction with those guidelines and a determination that there is an absence of current “disqualifying” information in this area. Of course, the polygraph (or computer voice stress analysis (CVSA)) is used to verify the veracity of applicants’ disclosures about their past illegal and immoral behaviors. Furthermore, the presence of past questionable behavior should not automatically terminate the hiring process.

It is unreasonable to disqualify every applicant who engaged in some delinquent behavior. As long as the negative past behavior falls within the agency’s guidelines, the important factor is for the hiring authority to determine the extent to which the applicant has matured and has the requisite commitment to ethical principles. Unfortunately, many background investigators and hiring authorities fail to connect the dots and, instead, discount the severity of applicants’ past delinquent behavior. For example, they may say something like, “He only had one fight his senior year of high school, only one undetected DUI in college, only called in sick to work when he wasn’t sick twice, and only missed a few car payments. Obviously, there’s no ongoing problem with aggression, alcohol, work performance or financial responsibility.” They have become so desperate to hire “qualified” applicants that they only see the behavior rather than the underlying personality characteristics that led to these behaviors. The reality is that those behaviors represent a pattern of poor impulse control and questionable ethics that does not appear to have abated even though it manifested in different ways. When the hiring authority is willing to offer employment to applicants who have a history of delinquent acts, it is essential for the agency to verify that a consistent pattern of mature and ethical behavior occurred since the last incident of questionable behavior.

The absence of disqualifying information in the area of moral character should not reassure police leaders that they are hiring applicants with strong integrity. You want to hire applicants who have a strong moral compass; with no disqualifying information obtained from the background investigation, you cannot be certain whether or not the applicant even has a moral compass. Instead, the hiring authority should demand affirmative evidence that every applicant who receives a conditional offer of employment has consistently displayed ethical decision-making. This can be accomplished when agencies adopt a screening-in hiring model [29]. Regarding integrity, background investigators should identify examples of impartiality, trustworthiness, and honesty, rather than just looking for tickets, arrests, and undetected criminal activity. Given today’s climate, it is particularly important for agencies to demonstrate that they are hiring applicants who have a proven track record of impartiality.

Although it is much easier for background investigators to identify a lack of integrity, they can be encouraged to spend the extra effort assessing the presence of integrity, as long as the agency’s hiring authority mandates screening-in as a priority. However, this requires some collaboration among the agency’s decision-makers to determine for what the background investigators should look. Each agency should define their own behavioral indicators of honesty, trustworthiness, and impartiality. The following is a partial list of positive examples of integrity:

- Applicants have owned up to mistakes:
  - They left a note after a parking lot fender bender.
  - They knocked on the neighbor’s door after breaking a window.
  - They earned money to repay their parents for damaging something.
  - They reported themselves to a supervisor/employer for an error.
- Applicants turned in found items (to lost and found or to the police).
- Applicants reported friends, neighbors, and/or co-workers whom they observed committing illegal acts.
- Applicants distanced themselves from friends engaged in illegal and/or delinquent acts.
- Applicants spent their free time in volunteer and charitable activities:
  - They were active in service clubs, e.g., Interact Club and Key Club, in high school.
  - They volunteered at soup kitchens, homeless shelters, and food banks.
  - They earned Eagle Scout and Girl Scout’s Gold Award.
  - They participated (and, ideally, promoted) in JROTC.
  - They participated (and, ideally, promoted) in their local police Explorers program.
  - They were involved in anti-bullying programs during middle and high school.
- Applicants demonstrated a commitment to helping others:
  - They obtained a Red Cross Lifesaving and/or First Aid certification.
  - They spent time in the Big Brothers Big Sisters of America.
  - Their high school jobs involved service, e.g., lifeguard, Little League umpire, etc.
- Applicants have been rewarded for responsible behavior:
  - They earned auto insurance discounts for safe driving.
  - They earned credit-line increases on their credit card.
- Applicants can demonstrate evidence of diversity in their friendships and support the claim with examples, such as attending pride parades and other multicultural community events.

The presence of these positive examples of integrity do not entirely negate evidence of delinquent behavior. Nevertheless, it is a positive sign if the delinquent behavior preceded these indicators of integrity. Moreover, for some applicants who sprinkled in some questionable behavior among acts of integrity, it will be up to the hiring authority to weigh the significance of the delinquent acts vis-à-vis the applicant’s character and maturity. Because everyone makes mistakes, the hiring authority has to determine whether or not the positives outweigh the negatives. Of greatest concern related to the moral risks of policing, however, is when the background investigation fails to identify a pattern of positive indications of integrity.

Finally, when it comes to assessing applicants’ moral compass, some have argued to include an integrity test during the hiring process [31,32,33]. However, these tests typically identify the risk of counterproductive work behaviors rather than measuring the applicants’ commitment to ethical principles. Additionally, there is a big difference between what applicants assert while completing a test and what they would actually do in the moment. Many people will convey that they know right from wrong when you test or interview them, but they lack the impulse control and judgment under pressure to do the right thing when faced with the actual decision. As the adage reminds us, actions speak louder than words. Those in a position to make hiring decisions should demand to see evidence of applicants’ prior ethical actions.

### 2.2. Training

Traditionally, discussions about police misconduct started with a common refrain: “It’s all about who you hire, who you hire, and who you hire.” This centered on the Mr. Goodwrench philosophy of “pay me now or pay me later,” whereby a bad hire can create a destructive ripple effect throughout the agency. Although this is still true about a bad hire, more recent evidence has shown that training—academy and in the field—plays a crucial role in whether or not officers will maintain a strong commitment to their ethical ideals [34]. In fact, in one study, following academy graduation, officers’ scores on an integrity scale decreased significantly after only one year in patrol [2].

When it comes to preventing negative impacts from the moral risks of policing, law enforcement leaders should not rely on hiring good apples. The job has a tendency to turn some good apples into bad apples and to turn even more good apples into bruised apples. Therefore, attention must be paid to training efforts that are designed to keep officers healthy and ethical. Instead of only a slogan on the side of a police cruiser, training should specifically define what the agency means by “to protect and serve” and should operationally ready recruits to do both. Like athletics, training needs to prepare officers for the physical challenges of the job, but their performance will suffer if training does not also prepare officers for the mental, emotional, and spiritual challenges of the job.

Before discussing training strategies designed to mitigate the moral risks of policing, it is important to say a few words about laterals. Because many police leaders see value in hiring laterals who have been trained by and worked at other law enforcement agencies, these employees should not be ignored when it comes to training. On one hand, lateral hires save the agency money, because they do not require academy training. This benefit, however, comes at a cost. Laterals bring with them the policing philosophy and culture from their previous agency. Some lateral hires also bring some baggage; they may have been disgruntled organizationally or impacted operationally by critical incidents at their previous agency. Rather than shirking the need to train lateral hires, every agency should implement a coherent plan to mentor and field train each lateral hire during the probationary period of employment. The goal is to address and “correct” the baggage, while socializing lateral hires to the culture of their new agency. Frankly, this plan should begin during the hiring phase with lateral applicants to ensure that they are exposed to the culture of the new agency (e.g., ride-alongs) and have realistic expectations of their new employer; they should understand that the grass is not always greener.

With an eye on preventing officers’ emotional and spiritual distress from the moral risks of policing and on reducing risks that can lead to officers’ misconduct, police leaders can embrace some innovative training techniques. Although the content of academy curriculum is set by state guidelines, the way in which the material is presented as well as additional co-curricular content can improve the ways in which officers are prepared for these challenging aspects of police work. The suggestions here may require adaptation to fit the specific parameters of your own academy and field training requirements. Agencies differ in the length of academy and field training. They also differ in the extent to which training is delivered in a para-military format. It is beyond the scope of this paper to address the ways in which a strict para-military format complicates efforts to mitigate the moral risks of policing. (Readers are referred to Blumberg et al. [1] and Blumberg et al. [35] for broader discussions of innovations in police academy training.) Nevertheless, training in the academy and continuing throughout officers’ careers can and should address ways to mitigate the moral risks of the job. To be blunt, there are unwanted, very negative consequences when training overemphasizes officers’ physical survival without simultaneously prioritizing their emotional, spiritual, and ethical survival.

#### 2.2.1. Minimizing Emotional and Spiritual Distress

Police leaders need to recognize that their officers will experience some amount of emotional and spiritual distress. When this is accepted as a by-product of routine police work, training can include strategies for officers to use, which will keep the distress manageable and prevent the distress from interfering with officers’ functioning. Training can provide officers with the mental preparation necessary to cope with this type of distress when it occurs. This involves attention to the service portion of “to protect and serve.”

You hired your officers, in part, because of their commitment to helping others and their strong desire to serve the community. This is a quality shared by the vast majority of police officers. Their compassion makes them good cops. Unfortunately, police training does not typically nurture this compassion and, in some cases, actually discourages it. Even when an agency’s culture reinforces officers’ compassion and service orientation, constant exposure to the worst that society has to offer leads many officers to experience compassion fatigue and other forms of emotional and spiritual distress. Therefore, police leaders have to establish academy and advanced officer continuing education training curricula that promote officers’ service orientation and provide tools for them to use in their battle against this distress.

One strategy is for police leaders to implement a trauma informed approach to policing, and, more broadly, to their organization. The Substance Abuse and Mental Health Services Administration (SAMHSA) [36] states the following:

*A program, organization, or system that is trauma-informed realizes the widespread impact of trauma and understands potential paths for recovery; recognizes the signs and symptoms of trauma in clients, families, staff, and others involved with the system; and responds by fully integrating knowledge about trauma into policies, procedures, and practices, and seeks to actively resist re-traumatization*.

(p. 9)

Trauma informed philosophies have been incorporated into many community service and treatment settings and have begun to be discussed in relation to policing, especially related to training officers to be more compassionate when interacting with victims of sexual assault [37,38] and with school children [39]. The approach trains officers to think (and ask) “what happened to you?” rather than thinking (or asking) “what’s wrong with you?” Trauma-informed policing is a method to prolong officers’ service-mindedness and sustain their compassion. It is an approach that generally elicits more compliance and appreciation from the public and can improve community-oriented policing efforts [40].

Additionally, when police leaders adopt trauma informed practices within the organization, officers will experience less emotional and spiritual distress. This begins in the academy by showing recruits that the organization: (1) knows that officers will be impacted by trauma; (2) will recognize when officers show signs of this impact; (3) will respond supportively; and, (4) will avoid re-traumatizing officers. If the academy provides a model of the trauma informed approach in the way recruits are trained (and supervised (see below)), it will be far easier for those recruits to effectively cope with their exposure to trauma when it inevitably happens. Likewise, modeling a trauma informed approach in the academy will provide recruits with the tools they need to use trauma informed tactics with people they encounter on the job.

A trauma informed approach to academy training does not mean that standards will be lowered or that recruits will be treated with kid gloves. However, utilizing this approach is a way to show support for your new employees (If you send your recruits to a regional academy, this is something that your agency should consider discussing with the academy director). Although the training may weed out some recruits who decide that policing is not the right career for them, such a decision should not be hastened by a lack of trauma informed practices. (It is beyond the scope of this paper to provide specific details about how to implement a trauma informed approach in your organization; anyone interested in learning more should contact the authors.) Some of your recruits have endured traumas in their past, including from childhood. With the proper trauma informed approach to their training, these recruits will be able to use those personal experiences to become excellent cops and strong assets to your organization. Conversely, a lack of trauma informed practices during training may result in re-traumatizing some recruits who then conclude that your organization’s culture will not provide a supportive work environment.

Beyond a trauma informed approach to training, one of the most important techniques to teach, which will minimize officers’ emotional and spiritual distress from the moral risks of policing, is compassion satisfaction, a term coined by Stamm [41]. Compassion satisfaction, in law enforcement, is the sense of gratification that comes from helping others, particularly those who have been victimized. Again, this is the feeling that most recruits seek when they enter the field with a burning desire to be of service to others. It is often difficult to maintain compassion satisfaction due to the overwhelming amount of suffering to which police officers are exposed, which leads to compassion fatigue, burnout, and other stress reactions [42]. Research has demonstrated that compassion satisfaction is negatively correlated with burnout and compassion fatigue among police officers [43,44]. Therefore, the goal for police leaders should be to find ways to assist their officers in efforts to maintain high levels of compassion satisfaction.

Training compassion satisfaction during the academy is like showing recruits how to properly wear their body armor. Without it, they become vulnerable to these moral risks. Specifically, as part of recruits’ stress inoculation training to prepare to successfully handle the various stressors that they will face on the job, academies should teach recruits how they can maintain a sense of pride, purpose, and accomplishment for the good work that they will do—despite the antipathy and depravity they will encounter. Training can spend more time emphasizing the positive work that officers will do and redefining what is meant by service. For example, in training scenarios when officers learn how to respond to a domestic disturbance, they should learn how to maintain a sense of satisfaction when making an arrest even when there is a belligerent victim (i.e., no one at the scene is appreciative of the officers’ efforts). In this situation, they can learn to take pride that their efforts may have prevented something far worse (e.g., a homicide) from occurring. Moreover, the academy should expand recruits’ definition of service beyond the help they offer victims who may not always be appreciative. Recruits can learn to take great satisfaction in their ongoing service to the community even when some members of the community seem to resent their presence.

More generally, other aspects of stress inoculation training will help police officers learn to insulate themselves from the moral risks that lead to emotional and spiritual distress. Techniques to boost resilience, to increase mindfulness, to strengthen spirituality, and to enhance overall wellness will better prepare officers to cope with these risks (see [27]). These efforts should begin during academy training. They should continue throughout officers’ careers as a routine component of advanced officer continuing education. However, it is actually counterproductive when agencies offer one-off trainings in these wellness areas without establishing an organizational infrastructure to reinforce what is taught. Without a broader culture of wellness and ethics throughout the organization, officers are not likely to continue to practice what they learn in these training classes.

#### 2.2.2. Deterring Misconduct

All police executives are concerned about officer misconduct. However, many fail to realize that certain organizational and operational practices actually lead to certain types of misconduct (see [2], for a review of organizational explanations for officers’ unethical behavior). The moral risks of policing that increase the likelihood of officer misconduct are associated with these organizational and operational practices. Specifically, certain types of misconduct are a by-product of moral disengagement [10], which disinhibits people from acting unethically. There are eight mechanisms through which moral disengagement occurs, and all eight can be demonstrated through the lenses of police training [2]. For example, moral disengagement increases when training does the following: underemphasizes a service orientation in favor of instilling a warrior mentality; suggests that the ends sometimes justify the means; perpetuates a strong Us (cops) versus Them (everyone else) attitude [45]; reinforces (even subtly) a code of silence among officers; and, promotes the view that some victims are sometimes responsible for their own misfortune. When officers develop these beliefs, they are more likely to be morally disengaged, which increases the likelihood that they will engage in some forms of unethical behavior.

The command staff in every law enforcement agency sets the ethical tone for the workforce. This starts during academy training, so there should be a resolute effort to prevent the development and spread of moral disengagement. Although officer safety is a paramount training objective, it should not overemphasize worst-case scenarios, which teaches officers to expect everyone they encounter to be a threat. Frankly, when officers are supremely confident in their self-defense, arrest and control, and defensive tactic skills, they will be comfortable with their ability to react appropriately once a true threat presents itself and less likely to interpret a threat when one is not objectively there. Therefore, the academy standards for these skills should remain quite high, if not set even higher. Then, training staff can shift the narrative during other training domains to emphasize community oriented policing techniques, ways to connect with and become part of the community, and how to maintain a compassionate service orientation.

Despite the colors of most police cruisers, the job is not black and white. There is much ambiguity in policing and moral relativity in society. At times, officers have to adapt to decriminalization (e.g., marijuana legalization) as well as to criminalization (e.g., helmet and seatbelt laws). Often, things are not as they first seem when officers arrive on scene. What is right and what is wrong may not be apparent. However, officers can always choose to act ethically. For example, officers’ use of discretion should be impartial and based on the situation rather than on some characteristics of the people involved (e.g., who gets a ticket and who gets a warning?). This becomes a challenge to those tasked with training new officers who need to learn how to do the job competently while being comfortable working in the gray.

Academy training should focus on preparing recruits for the moral ambiguity that they will face on the job. It just will not work to tell recruits what to do in some of these situations. Instead, recruits have to be taught how to think for themselves. Training can prepare recruits by having them identify and solve complex moral dilemmas [46,47], which they are likely to encounter on the job and off-duty. Veteran officers can describe the choices they made when faced with ethical dilemmas, but the recruits need to gain experience figuring out for themselves how they will decide on the “correct” course of action in ambiguous situations. This should include discussions among recruits about why certain choices might be better than others in various situations where there is not one clear decision to make. Similarly, the academy should highlight case studies of ethical breaches so that recruits learn what will happen if they make the ethically wrong choice. The disciplinary outcomes of these cases of misconduct may be agency-specific and more difficult to convey in a regional academy. However, the case studies presented should represent agencies from where recruits in the regional academy were hired.

It is not always easy for police officers (or anyone for that matter) to know what to do in an ethically ambiguous situation. Sometimes, mistakes will happen. (The authors differentiate between mistakes made because of a lack of knowledge or skill and those made because of uncertainty in a morally ambiguous situation.) Academy training can deter future misconduct by helping recruits learn not to beat themselves up over a mistake. Of course, this is easier said than done, because some mistakes can have tragic consequences. However, recruits can be trained to face the consequences of their actions with humility and integrity. Conversely, if training implies that there is no room for any mistakes or that recruits will face termination regardless of the mistake, then they are more likely to cover up or lie for themselves and each other. Additionally, when the training environment creates an atmosphere in which mistakes are not tolerated, these recruits will become officers who are unable to cut themselves some slack after a blunder. This lack of self-forgiveness can lead to a deterioration of officers’ commitment to ethical principles (e.g., “I’m no good, so what’s the use?”). It also can contribute to officers’ experience of moral injury [17], where intense feelings of guilt or shame develop after officers do something (or fail to do something) that violates their moral principles. Therefore, it is incumbent upon the academy training staff to create an environment where mistakes are handled with appropriate and proportionate discipline. From an educational perspective, especially when talking about mistakes associated with ethical dilemmas, recruits should learn that mistakes are learning opportunities and that the academy is the place to make them.

Another way in which the academy and advanced officer continuing education can deter misconduct is by implementing emotional intelligence training. This is relevant because research has demonstrated that people with higher emotional intelligence make fewer unethical decisions [48]. Moreover emotional intelligence improves police officers’ job performance [49]. This is not simply an inherent trait that officers have or lack. Recent research demonstrated the efficacy and benefits of emotional intelligence training with police officers [50]. The training should be experiential and designed to enhance officers’ emotion regulation and emotional competence in their interactions with the public as well as with fellow officers, superiors, and all members of the agency [20]. The goal is to improve recruits in the four areas of emotional intelligence: self-awareness, self-management, social awareness, and relationship management [51]. It can be argued that it is as important for academies to increase recruits’ emotional intelligence as it is to prepare them for every other aspect of the job [52]. This is especially true when it comes to helping officers immunize themselves against some of the moral risks of policing.

### 2.3. Supervision and Promotional Practices

It does not really matter how well recruits are trained in the academy if police leaders do not reinforce what was taught once these new officers hit the streets. Furthermore, agencies’ command staff should not assume that the academy adequately covered everything that they want their officers to know regarding the moral risks of policing. Some of this can be handled through advanced officer continuing education. However, much has to be dealt with through field training, supervision, and performance evaluation practices. This is a vital area for police leaders to consider when focusing on retention efforts.

The first step is to ensure that the agency’s Field Training Officers are competent and motivated. FTOs are your new officers’ introduction to the culture of the organization, especially for officers who were trained at regional academies. Beyond helping new officers adapt what they learned at the academy to the unique customs and procedures of the agency, FTOs prepare new officers for the moral risks that they will face. The FTO sets the tone for how emotional and spiritual distress will or will not be handled. Ideally, FTOs have learned how to emphasize officer wellness and to take a mentoring role with their trainees rather than fostering fear through intimidation. Likewise, when it comes to mistakes and the moral risks that can lead to misconduct, FTOs are the role models. New officers, like sponges, will absorb everything they see and hear from their FTOs, including attitudes and behaviors that are representative of moral disengagement. Therefore, police leaders who are concerned about the moral risks of policing will start with a critical analysis of their FTO program, including how FTOs are selected, trained, and supervised.

For now, let us assume that every officer was field trained by excellent FTOs. The focus, then, can shift to broader supervision and promotional practices geared to mitigate the moral risks of policing. The first step to minimize officers’ misconduct and their emotional and spiritual distress is to increase efforts to detect early warning signs of problems. This requires front line supervisors to be trained in what to look out for as well as an infrastructure that contains steps for them to take when they see something. Those sergeants need to be supported by their lieutenants who need to be supported by their captains and so forth all the way to the top. An organizational culture of wellness and ethics contains policies and procedures for all supervisors to follow regarding early detection and intervention, which is discussed below.

One aspect of a culture of wellness and ethics is the organization’s promotional practices. For many agencies, these practices are at least partially established by your state’s POST guidelines, state and local labor codes, HR policies, and, in some cases, union requirements. Independent of these rules, however, is the opportunity for police leaders to inject their own standards into the agency’s promotional practices. Fundamentally, promotions should not be based solely on the quality of the individual’s work in their current position. This, in no way, provided an indication of how well they will perform at the next level. More bluntly, many great cops make terrible supervisors. The promotion should be based on merit and a demonstrated ability to be the kind of leader who will uphold the organization’s culture of wellness and ethics. Interview questions during the promotional process should require candidates to discuss their philosophy and to provide solutions to hypothetical scenarios related to the moral risks of policing. Regarding prevention of the consequences of the moral risks, these scenarios should describe officers in various stages of those negative impacts: Can the candidate identify early warning signs of distress and, if so, what is the action plan? How will the candidate respond as signs of distress intensify? To achieve this, police leaders should consider implementing a mandatory training class for all supervisors and for all officers who will participate in the promotional process. The class can be tailored to the specific practices of each organization and provide attendees with a certificate of completion, which would be a necessary component of every candidate’s promotional package.

For now, let us assume that every supervisor throughout the organization was promoted because of their commitment to officer wellness and ethics. The focus, then, can shift to specific steps that can be taken to prevent the negative outcomes of the moral risks of policing.

#### 2.3.1. Minimizing Emotional and Spiritual Distress

The importance of compassion satisfaction in mitigating officers’ emotional and spiritual distress was described in some detail earlier. Compassion satisfaction allows officers to continue to find purpose and meaning in the work they do despite all the pain, suffering, and antagonism that they regularly encounter. It should not be too hard for police leaders to imagine, then, how difficult it is for officers’ to maintain a sense of compassion satisfaction if they do not feel appreciated by their employer. Therefore, the first step for supervisors to take to help minimize officers’ emotional and spiritual distress is to establish routine methods to show appreciation and to boost morale. The organization can accentuate the positive by highlighting officers’ good deeds, letters of appreciation from grateful citizens, and the under-the-radar incidents of de-escalation that prevented an incident from going sideways. This should be a regular part of every pre-shift rollcall, so officers have these positive reminders as they hit the streets. Some chiefs record a video message once a month or every quarter, which is played before each shift. The message should highlight officers’ positive actions and remind them about the good they are doing. Even better, the chief and/or assistant chiefs should make regular visits to deliver these positive messages in person. Officers derive benefit from hearing their leaders explicitly talk about compassion satisfaction and from seeing them highlight the good work that officers do. This also should be done on a more personal level by each officer’s direct supervisor. It will be easier for officers to feel good about their work if their supervisor is skilled at giving pats on the back rather than just pointing out mistakes or focusing on areas that need improvement.

Many officers feel as though they are a metaphorical island while working their assigned beat. Such a feeling is a breeding ground for compassion fatigue. Consequently, another way to minimize officers’ emotional and spiritual distress is to increase a sense of teamwork. It remains to be empirically tested with police, but a recent study demonstrated the value of “interprofessional collaboration and transformative leadership” on reducing symptoms of secondary traumatic stress ([53], p. 7). Additionally, burnout and compassion fatigue have been shown to be inversely related to teamwork [54,55].

Therefore, supervisors should try to reduce competition and increase collegiality and camaraderie among officers. For example, organizations can eliminate officer-of-the-month/quarter/year recognitions and start highlighting the accomplishments of a particular squad or division. This is particularly critical, yet more difficult, on smaller departments that do not have many officers on-duty on any given shift. The sense of camaraderie may not be able to be derived from teamwork with squad mates. Instead, supervisors can initiate off-duty opportunities for officers to connect and to bond and should ensure that there are no on-duty competitions among their subordinates. For example, regardless of the size of the agency, supervisors can build teamwork and increase compassion satisfaction by organizing teams of officers to join community-based charity events, such as 5K runs. It is important that those events involve activities in which all of the officers are comfortable participating.

Another strategy to minimize emotional and spiritual distress is for supervisors to openly confront the moral risks that can cause it. Whenever officers are exposed to something horrific or are unable to save a victim and whenever supervisors have to implement an unpopular policy that might prevent officers from doing something they think would help people, supervisors should explicitly acknowledge officers’ feelings of sadness, frustration, and powerlessness. Conversely, when supervisors say things like, “It’s just the way it is,” “If you don’t like it, you can always quit,” or “You better get used to it, because it comes with the territory,” they are actually fostering compassion fatigue. Knowledge is power, so supervisors should help their subordinates understand that some emotional and spiritual distress is inevitable but that it does not have to interfere with officers’ job satisfaction. Simply put, supervisors should not ignore officers’ distress and should do what they can to emphasize teamwork and their officers’ positive accomplishments [56].

#### 2.3.2. Deterring Misconduct

Many police executives are explicitly committed to reducing unethical behavior among their ranks. This has become both a sincere objective and the politically correct thing to say. Unfortunately, there is quite a chasm between talking about preventing police misconduct and successfully lowering the incidence rates of officers’ transgressions. Nevertheless, frontline supervisors have the most important role in the organization to deter officer misconduct. They are responsible for consistently conveying the moral tone of the organization. For example, a retired 30-year law enforcement veteran who is a colleague of the first author related something that occurred in the first few months of his career: “There was a call with six or seven of us at the scene. Our sergeant got us all together afterwards and said, ‘Before any of you write a report, make sure to get your stories straight.’ He was more concerned about how it would look than he was about everyone telling the truth.” This type of supervision condones misconduct in the moment, which inevitably increases the probability that at least some of those officers will increase the frequency and severity of future unethical acts [13].

The association between frontline supervisors’ actions and officers’ misconduct cannot be overstated. As mentioned in the training section, officers will act ethically if they have learned how to resolve complex moral dilemmas and how to make the ethically right choice in ambiguous situations. Once in the field, it is imperative that officers’ FTOs and frontline supervisors continue to reinforce this training and to consistently encourage officers to think for themselves. It appears that at least certain forms of misconduct, especially those associated with moral disengagement, will be deterred when officers believe that their decisions have been made freely and were not, simply, because they were following supervisors’ orders.

Some recent research helps to explain this phenomenon. Although it remains to be tested in a non-laboratory setting or with a law enforcement sample, fMRI brain imaging demonstrated a relationship between following orders and a lack of empathy to others’ pain [57]:

*Here, we have shown that when people accept to comply with the orders of an authority, the neural response associated with the perception of pain felt by another individual is reduced in comparison with being free to choose which action to perform…We also show that participants’ negative feelings and neural guilt signatures were reduced when they comply with orders. These results highlight how obeying an order relaxes our aversion against harming others, despite still being the author of the action that led to the pain*.

(p. 24)

In the least, such findings should give police executives pause. Para-military style training and supervision in which officers’ are conditioned only to follow orders diminishes officers’ sense of autonomy and self-efficacy. This may increase the likelihood that officers will feel less remorse after a moral transgression. More importantly, there may be neurological reasons why having greater independence of thought and action would decrease the likelihood of officers committing a moral transgression in the first place. This is an exciting area for future research.

On a more practical level, there are some tangible steps that agencies can take, which would enable supervisors to deter misconduct. The first step is an extension of what was mentioned in the training section about mistakes. Officers who fear making mistakes are likely to be more worried, which will make them tenser. Paradoxically, this will cause officers to make more mistakes [58]. Part of an agency’s culture of wellness and ethics is for supervisors, throughout the chain of command, to adopt the mindset that mistakes, even occasional lapses in ethical decision-making, will occur. This normalization process will help to destigmatize the behavior; officers, then, are able to perform at peak levels without the interference of anxiety, which will prevent certain transgressions from happening (see below for how agencies can respond effectively when mistakes do happen).

Another strategy to deter misconduct is for supervisors to do a better job of championing officers’ ethical decision-making. It is well established that positive reinforcement works and that it works much better than punishment when it comes to shaping behavior. If supervisors want officers to remain steadfastly committed to ethical principles, it is important to support and reward officers when they demonstrate excellent integrity and ethical behavior. Establish a formal way to highlight ethical decision-making, especially when officers are faced with tough choices, but make the ethically correct decision. This is where some friendly competition may not be a bad idea, such as a prize to the ethical officer of the month/quarter/year. Supervisors should be incentivized to do this by awarding similar prizes to the ethical sergeant/lieutenant/captain of the month/quarter/year as well. When police leaders increase the positive attention to ethical behavior, it can have a generalizing effect throughout the organization, because officers will serve as good role models for each other.

This leads to the important point that supervisors are role models. Officers should never get the sense that a supervisor is conveying a “do as I say, not as I do” attitude. Instead, they have to demonstrate an unwavering commitment to ethical decision-making if the organization hopes to deter officers’ misconduct. Along with ensuring that the organization has supervisors of high integrity, it is essential that supervisors model fairness and consistency. The consequences for misconduct should be clearly articulated by the organization and completely understood by every officer. However, when supervisors treat their subordinates disparately, the officers lose incentive to act ethically. It is essential for fairness and consistency to be demonstrated throughout the chain of command so that everyone at all ranks sees the importance of integrity and sees that everyone, regardless of rank, is held accountable to high ethical standards.

## 3. Wellness Efforts

A quote of uncertain origin is “Hurt people hurt people,” which underscores the importance for law enforcement organizations to become trauma informed. Operationally, this philosophy will help officers to avoid unnecessary escalations with community members who are suffering the effects of traumatization. Organizationally, however, a trauma informed approach recognizes that officers who are experiencing the negative impacts of the moral risks of policing are more likely to act in ways that hurt themselves and others. Depending on how this manifests (e.g., guilt, anger, anxiety, exhaustion, etc.), those actions can cause a variety of problems for the officers and for the organization. Therefore, it is essential for police leaders to implement comprehensive officer wellness initiatives.

The important first step is for police leaders to conceptualize wellness as a perishable skill [2,21]. For example, although officers have a driver’s license when hired, agencies never assume that they will be proficient enough behind the wheel to simply skip emergency vehicle operations training and regularly scheduled driving requalifications. In fact, most states mandate a minimum number of continuing education hours of training in skills that are considered to be perishable. Likewise, officers have to be trained to cope with the stresses of police work and should have resources readily available to keep them functioning at peak levels. This is a proactive approach to wellness that focuses on the prevention of stress-related problems, which include lapses in ethical decision-making. Therefore, efforts to maintain wellness are critical to deter officer misconduct.

There are many ways for organizations to approach officer wellness. Some organizations maintain a reactive approach, which provides only intervention services such as an Employee Assistance Program (EAP) or contracted mental health providers for officers to contact when they want help. This would be like requiring firearms training and regular requalification only after an on-duty shooting. The reactive approach conveys a disturbing message to the agency’s officers that maintaining wellness is not very important to the command staff, but that there are services available when officers start to experience problems. Conversely, when organizations take a proactive approach to officer wellness, command staff demonstrates that they are committed to keeping their officers healthy.

The proactive approach to wellness should include a dedicated Wellness Unit [20], which should be staffed with at least one sergeant and two officers. Minimally, the agency should have a designated Wellness Officer at the rank of sergeant or higher. Although the Wellness Unit is available to officers who are struggling and Wellness Officers respond to on- and off-duty critical incidents, the main focus is on promoting wellness from the time officers begin in the academy through their tenure with the organization. Wellness Unit staff should consider ways to implement activities and initiatives that promote the power of POWER (Police Officer Wellness, Ethics, & Resilience) [59,60]. This work can include monthly newsletters, videos played at rollcall, and various continuing education wellness-related classes. Wellness Officers also can foster improved morale by sponsoring off-duty activities and participation in community and charity events. The officers who are assigned to the Wellness Unit can serve as liaisons between the organization’s psychological service providers and the agency’s employees to deliver health-promotion strategies and ways to emphasize wellness and ethics. The Wellness Unit also is available to officers who want to prevent problems from developing at home. The Wellness Officers can provide resources to officers’ family members and assistance when it comes to successfully juggling home-work stressors. The establishment of a Wellness Unit explicitly communicates that the agency is committed to maintaining a culture of wellness and ethics.

## 4. Intervention Efforts

Despite the best efforts of law enforcement leaders, some officers will continue to experience emotional and spiritual distress. Furthermore, unfortunately, some officers will continue to engage in misconduct. The moral risks of policing can be mitigated, but not eliminated. Therefore, intervention efforts, some of which may be considered rather innovative, must be considered. Given the current climate of policing in the United States, it may be time to get creative when designing methods to address this complex problem. In the end, these suggestions combine to create procedures designed to enhance officer retention.

The discussion begins with ways to detect early warning signs. It is always easier to intervene when someone is, metaphorically, whispering to you than when they are yelling in your face. For one thing, it is hard to remain compassionate when someone is yelling at you. Furthermore, by the time they are yelling, they will be less receptive to any help that is offered. However, it is much more difficult to hear the whispers. Therefore, effective early detection interventions begin by improving the listening skills of supervisors, FTOs, and peers throughout the organization. Early detection should be an organizational, all-hands endeavor. This section also provides several intervention ideas for when the “yelling” begins. Regarding the moral risks that lead to emotional and spiritual distress, the strategies focus on officers’ overall health. Conversely, regarding the moral risks that increase the likelihood of misconduct, the strategies focus on the overall “health” of the organization.

### 4.1. Emotional and Spiritual Distress

Early detection of officers’ distress is not possible when people do not know for what they are looking. In addition to preparing officers to be aware of their own emotional and spiritual distress, everyone in the organization should be trained to identify the early warning signs. Interventions are generally more effective when they begin at the first indication of a problem. This requires familiarity with the individual’s baseline or “normal” functioning, which depends on how well acquainted you are. Co-workers often know each other well enough to recognize a subtle change, but may not understand that such changes could be an early warning sign of distress. Frontline supervisors have to make the effort to get to know their subordinates on a personal level in order to recognize subtle changes [42]. The same is true for officers up the chain of command. The better you know someone, the easier it is to notice when they seem a bit off (i.e., not themselves).

There are several categories of signs of distress: physical, emotional, cognitive, and behavioral. Before describing them, however, it is important to mention that these indicators also could just mean that the person is having an isolated bad day. The signs of distress take on greater significance with longer duration and when they occur more frequently and/or with greater intensity.

When people are struggling to cope, the distress may manifest first physically, due to a compromised immune system [59]. These physical indicators may not be overtly apparent to most people, but supervisors and close peers would be able to notice them. For example, the person is not just fatigued, they are exhausted. They typically are rarely sick, but now seem to be unable to shake a cold. They may complain about or seem preoccupied by a physical ailment (e.g., that old back or knee injury), which usually does not seem to bother them. Headaches, digestion issues, insomnia, and weight changes also may be early signs of distress. To cope with the physical symptoms (rather than with the underlying emotional or spiritual distress), many people turn to self-medication. This could be an increase in the use of caffeine, alcohol, or tobacco. It also could be the more frequent use of antacids or analgesics (e.g., aspirin, Tylenol, and Advil). Self-medication also can be turning to comfort foods and overeating. It is especially important for supervisors to keep track of their subordinates’ use of sick leave. It is very noteworthy if someone who rarely uses any starts missing work because of illness.

Not surprisingly, there are emotional indicators. Some of these are more easily noticed by others than the physical indicators. But, again, it depends on how well you know the person, so that you can detect a change from their baseline mood. Do not discount it when you see someone who appears more glum, nervous, or irritable than usual. Likewise, more intense suspiciousness, apathy, or guilt should be addressed. For supervisors, be alert when a subordinate begins to show more bravado. This should not be confused with confidence; it reflects a level of pretentiousness that can lead to recklessness. Perhaps most importantly, beyond an early warning sign, if anyone ever gets even the vaguest idea that a fellow officer might be thinking of hurting him- or herself, do not ignore the feeling; stay with the individual and call for some backup (i.e., a mental health professional).

The cognitive warning signs can confound supervisors and, for this reason, are fairly easy to spot. They reflect the fact that the person who is starting to manifest signs of distress is distracted. The cognitive indicators include inattentiveness, poor time-management, missed deadlines, forgetfulness, scattered or off-topic thought processes, appearing overwhelmed, and general “wheel spinning” (e.g., making lists, but accomplishing little; frequent do-overs).

Some of the behavioral indicators are fairly easy for close friends and co-workers to notice. Often, when people begin to struggle, they will withdraw and isolate themselves. The officer who routinely attended after work get-togethers, weekend barbeques, and office-related parties starts making excuses and never shows up. Conversely, the officer continues to attend functions, but it is evident that he or she is drinking a lot more than usual and, maybe, far too much. This goes along with officers who were very conscientious, but begin to display some carelessness and even recklessness on-duty and off-duty.

Individuals who begin showing these signs may not be aware that they could have a problem with emotional or spiritual distress. That is why it is so important for those close to the person to spot these early indicators and do something to intervene. However, when someone knows what to look for, there is no guarantee that they will do something once they notice something. Two common refrains are “I don’t want to piss him off” and “I don’t want to do anything that could mess with her career.” Unfortunately, when people fail to act when they notice an early warning sign, the behavior is likely to get worse until it may be too late. It is an essential component of an organization’s culture of wellness that there will be no reprisal against officers if they or their family members reach out for assistance.

Now, when someone notices something and makes the decision to act, the organization must have an infrastructure in place. This is essential for others to be able to intervene successfully, and also for employees to be able to self-refer when they begin to recognize that they are having some struggles. Everyone needs to know what to do if they are concerned about the well-being of a colleague. This is one of the foundations of an organization that has established a culture of wellness. Not surprisingly, two of the key indicators of an agency that has not prioritized officer wellness are: (1) officers are confused where to turn when they are worried about a colleague; or, (2) officers are reluctant to utilize department resources when they are concerned about a co-worker because of a lack of trust in those resources. Conversely, organizations with a strong culture of wellness provide numerous options for their employees.

A logical and fairly common place to go to intervene when concerned about a colleague is the agency’s mental health provider. If the agency has an in-house psychologist, officers will be familiar with and, hopefully, comfortable talking to him or her. On the other hand, if the organization’s psychological services are delivered by an outside contractor, there may be less familiarity and, therefore, less comfort seeing that provider. More importantly, however, the mental health professionals need not be the first stop when dealing with the aforementioned early warning signs. Of course, consulting the department psychologist is never a bad idea, especially when concerned about something potentially serious. But, if the concerns are about early warning signs, a suggestion to see the shrink may result in the officer thinking that everyone is making a mountain out of a mole hill. Also, there are other, very appropriate options to consider when one has detected early warning signs of officers’ emotional and spiritual distress from the moral risks of policing.

Two very effective early interventions when it comes to the moral risks are peer support and an active chaplaincy [28]. Although peer support officers and department chaplains are important resources following critical incidents and to provide assistance to officers and their families when things are more urgent and/or severe, they should be utilized, also, at the earliest indication of officers’ distress. Chaplains are an important resource to help officers who are experiencing emotional or spiritual distress and to offer strategies to enhance compassion satisfaction. Officers, even those who are not religious, should be encouraged to contact one of the department chaplains for some guidance and support at any time they start to feel some distress. (Agencies without a chaplaincy program are referred to Creighton and Kaye [28] for some important information to consider before accepting any members of the clergy to assist your officers.)

Peer support also is an important resource as an early intervention. This happens all the time informally, to varying degrees of success. Most co-workers mean well, but many simply abdicate the role by referring the distressed officer to a professional or they say or do something that inadvertently makes things worse. Peer support in this context refers to officers who have been trained and certified to take on this collateral duty [28]. Many volunteer because they have successfully handled various police-related stressors and can be very helpful to officers who are experiencing similar challenges. Peer support officers are relatable and provide a realistic “light at the end of the tunnel” for officers who are struggling. The peer support program can fall within the purview of the agency’s Wellness Unit, so that peer support officers are not just seen as someone who shows up at a crisis. They should take part in proactive and preventative wellness activities, so that officers are familiar with and comfortable engaging members of the peer support team even during the early signs of distress.

Another intervention technique is family engagement. The agency only sees officers when they are on-duty. Some officers who are struggling are in denial, do not think there is anything wrong, and are able to put on a good façade while at work. Conversely, they let down their guard at home. Family members see the change, but may not know where to turn or how to help. Moreover, the officers’ distress may stem from something at home [28] or from something that occurred off-duty. If these issues are not dealt with early, they will likely intensify and cause disruptions in officers’ work performance. The agency’s culture of wellness should extend to the family, so that they are well acquainted with resources available to them when they are worried about their officer. Chaplains should be available to officers’ spouses. Additionally, under the auspices of the Wellness Unit, the agency could utilize a Spouse Auxiliary. Among many activities in which members of the Auxiliary may be involved, providing some guidance and support to the family members of officers should be at the top of the list.

Self-assessment and self-referral are additional intervention tools. The organization’s culture of wellness and ethics promotes the importance of knowing when to get some help and where to go when you want it. This requires a two-step process on the agency’s part. First, the Wellness Unit and other agency initiatives, such as Chief’s monthly videos, should teach officers about the signs and symptoms of emotional and spiritual distress, compassion fatigue, burnout, moral injury, etc. This serves an important normalization process with the goal of reducing stigma associated with these struggles. Secondly, everyone needs to know where to turn to receive some support. Members of Peer Support, Chaplaincy, Spouse Auxiliary, Psychological Services, and, of course, the Wellness Unit should intermittently attend rollcalls to increase officers’ familiarity and comfort level with the folks to whom they and their family can turn when experiencing some distress.

There are times when the early warning signs are missed and more intense interventions are required. This is when officers’ performance has clearly been impacted by their distress or when there are obvious signs of psychological disturbance, such as anxiety, depression, or PTSD. The agency’s obligation at such times may be to require a fitness-for-duty evaluation, which is strictly a risk-management (i.e., vicarious liability) decision. However, the agency also should have supportive resources available to the officer. Most commonly, agencies have in-house or contracted mental health providers to intervene. Additionally, the agency’s Wellness Unit should make toll-free police hotline numbers available so that officers can pick up a phone and talk to someone if they are in crisis (e.g., copline.org), but are uncomfortable contacting a department mental health provider. For the more severe situations, the Wellness Unit also should establish a relationship with one or more of the reliable inpatient treatment centers that are dedicated to treating first responders who are suffering from things like alcohol/substance dependence, severe depression, PTSD, suicidality, etc. There may not be one of these clinics geographically near your agency, but they treat first responders from around the country. Beyond just having the phone number, it is important for Wellness Unit staff to be familiar with the availability, reliability, and admission criteria of the selected clinic(s). They should ensure that the facility accepts the medical insurance carried by your agency’s employees. Moreover, they should have the names of specific staff at the facility to contact who will facilitate admission of your officers, because some of these situations are very time sensitive. Although all the work on early detection of distress will be beneficial, organizations with a strong culture of wellness are prepared to handle things when more extreme interventions are necessary.

### 4.2. Misconduct

Effective interventions after an officer makes a mistake are one of the foundations of a healthy organization. The first step, as mentioned previously, is early detection. Oversight and discipline will help to prevent some misconduct, but also are critical components at the first sign of misconduct in order to deter a repeat or an escalation of the behavior. (The authors understand the extent to which progressive disciplinary actions may be established and regulated by outside factors (e.g., state labor code, city HR policy, and union CBA) and that changes in disciplinary procedures may be very difficult to implement. Rather than viewing the suggestions as idealistic, it is hoped that they are seen in the context of an organization’s culture of wellness and ethics with an eye toward engaging all stakeholders in negotiations to improve the way in which misconduct is handled.) Unfortunately, in agencies without a strong culture of wellness and ethics, supervisors may react to “minor” transgressions by sweeping them under the proverbial rug. In other cases, supervisors may ignore the misdeeds of some officers, but discipline similar behavior committed by other officers. This is both ironic and troubling when officers similarly misuse discretion on the street; remember, supervisors are the role models of ethical and unethical behavior. These disparate and inconsistent supervisory practices will lead to more misconduct. Therefore, it is strongly recommended to shift the disciplinary focus to a philosophy of corrective counseling, which will enable agencies to respond to officers’ mistakes in a consistent and constructive manner [2].

The most apt analogy is a penalty in sports. Healthy agencies recognize that mistakes, including some acts of unethical decision-making, are bound to happen. They establish a hierarchy of responses. In ice hockey, there are two-minute minor penalties, four-minute double minor penalties, five-minute major penalties, and misconduct penalties in which the offending player is out for ten minutes or ejected from the game. Football has a similar hierarchy of interventions for various misdeeds. In any organization, as in these examples, it is essential for the behavior to be flagged by a frontline supervisor. Once a penalty is called, officers should know exactly what will happen. There should be no wiggle room: “If I do that, this is the consequence.” Organizations can develop, with their police union officials, a sort of penal code, so that everyone understands which misdeeds are infractions, which are misdemeanors, which are felonies, and which are fireable offenses. A culture of wellness and ethics promotes the notion that the organization cares about officers and will continue to support them no matter what happens (including termination). The idea here is that there is nothing personal about the discipline, which is exactly the message that the agency wants officers to convey in their dealings with the public, whether it is giving a traffic citation or having to use force to make an arrest.

In relation to the moral risks that increase the likelihood of misconduct, supervisors have to be trained and incentivized to identify and to respond to even the smallest examples of misconduct. Each instance is an opportunity to counsel the officer. Did they know what they were doing? Did they know it was wrong? Did they do it intentionally? Is there something that could be done to help them not do it again? Supervisors should keep track of officers’ ethical behavior. For example, agencies can include “categories of objective, observable evidence of ethical behavior on officers’ annual performance evaluations. Officers whose ratings fall short of these standards associated with positive incidents of ethical behavior would be targeted for early intervention efforts” ([2], p. 11). In other words, a lack of strong ethical behavior should be enough to warrant some corrective counseling even when there has not been overt misconduct. That counseling may be little more than some encouragement, with clear examples, to demonstrate more evidence of ethical behavior. Or, an early intervention could be mandated attendance at an ethics and prosocial behavior workshop put on by Wellness Unit staff.

As misconduct increases in severity, interventions have to become tougher. Some have argued that harsh discipline is a deterrent [24], but it also is a vital response when officers engage in unethical behavior. The behavior should dictate the response. Mitigating factors, including how well officers have performed throughout their career before the misdeed, should not be considered. This takes the discretion out of the equation and treats everyone in the organization, regardless of rank, equally. Regardless of the transgression and the subsequent discipline, organizations with a strong culture of wellness remain compassionately supportive of officers who have acted unethically. In fact, some officer misconduct is the result of emotional or spiritual distress. The behavior still deserves the disciplinary response, but the person deserves support and access to appropriate resources.

Officers may attempt to excuse their actions by claiming a temporary psychiatric disability. In some cases, officers file a worker’s compensation claim after an act of misconduct with the intention of shifting responsibility for the mistake. Officers who are suffering from various conditions are more likely to do something wrong. For example, those with substance abuse problems are more likely to get stopped for DUI, abuse sick leave, or come to work with a hangover. However, many officers suffering from similar conditions do not get in trouble. They either avoid misconduct despite their struggles or they get some help for the problem before doing something that would result in discipline. This is why disciplinary outcomes should not be influenced by mitigating factors. Officers should know ahead of time exactly what the consequences for each transgression will be and understand that nothing will alter that outcome. At the same time, officers may have legitimate work comp psychiatric injuries. Despite the city or county’s role in litigating those claims, the organization should not be viewed as adversarial in those proceedings. Injured officers, even those who receive discipline for misconduct, should continue to feel supported by their employer and encouraged to utilize available department resources, like the Wellness Unit, Peer Support, and Psychological Services.

Agencies are usually prohibited from publicizing the outcomes of officers’ disciplinary actions for privacy reasons. This is a missed opportunity to utilize intervention procedures for prevention purposes. Names can be redacted, but the organization might consider a monthly report of transgressions and subsequent disciplinary outcomes. It could deter officers from committing a “minor” infraction, like accepting a small gratuity, if they saw that a colleague received, say, a day off without pay for such misconduct. These efforts may require the buy-in of the union. The question becomes to what extent will the union agree to share the responsibility to deter and to intervene following officers’ unethical behavior? Unfortunately, this is rhetorical, because most union leaders seem willing to go to great lengths to protect their members from disciplinary actions even after indisputable acts of misconduct. Nevertheless, everyone associated with the organization should demonstrate a commitment to officer wellness and ethics and actively participate in some of these interventions. This is a critical step, also, when it comes to the agency’s legitimacy among members of the community. One hopes that union leaders would want to improve the community’s view of their officers as much as the organization’s leadership does, since this ultimately impacts officer safety. Perhaps this becomes a topic of negotiation before the next CBA is agreed upon.

This raises another intervention strategy, which may require the blessing and cooperation of the union. Organizations should consider establishing alternatives to traditional discipline in response to certain acts of officer misconduct. One suggestion is for organizations with a strong culture of wellness and ethics to adopt a restorative justice paradigm. “Restorative justice offers those responsible for wrongdoing the opportunity to accept responsibility, regain self-respect, and be reintegrated into the social settings whose norms they have violated without having to bear the stigma of their offense” ([61], p. 626). This would help to shift the narrative about misconduct away from the “bad apple” perspective to the view that officers can make amends and be forgiven for (most of) their transgressions. Many police leaders fail to see the extent to which officer misconduct is an indictment on the agency. Incorporating restorative justice practices says to officers that we still value you even if you make a mistake. However, after a mistake, there will be steps that officers will need to take to repair their reputation and the reputation of the organization, which their misconduct injured. Again, restorative justice works only on transgressions that would fall at or below the lowest “felony” misdeeds in the agency’s “penal code.” The more serious misconduct, such as lying, will irreparably injure officers’ reputations within the organization and the broader criminal justice system.

## 5. Leadership and Maintaining a Culture of Wellness

Establishing and maintaining a culture of wellness and ethics requires leaders to have and to consistently display moral courage. Moral courage, most simply, is the willingness to do the right thing even when it is not popular and even at the risk of some negative consequences ([62], p. 511). There are two aspects of moral courage related to the agency’s culture of wellness and ethics, both of which reflect the importance for officers to see moral courage from their leaders [63]. The first aspect relates to officers’ work behaviors. If leaders want their officers to do the right thing, no matter what, when interacting with the public, then the leaders have to model moral courage. For example, in one study that used a non-police sample, it was found that “courageous leaders are able to inspire and encourage followers to act prosocially” ([64], p. 597). Within police agencies, although this remains to be empirically tested, it can be assumed that leaders who demonstrate moral courage will have officers who are more likely to maintain a strong service orientation, despite the challenges. In practical terms, leaders with moral courage have their subordinates’ back; officers are confident that they will never be thrown under the bus. This does not imply that misconduct will be ignored. Rather, it means that the misconduct will be fairly and consistently handled according to the disciplinary guidelines and not influenced by public or media attention or by internal organizational pressures.

The second aspect of moral courage is organizational and relates to the ways in which police leaders embolden courage to be displayed within the agency [65]. A culture of wellness and ethics encourages employees to speak up and to offer solutions when a problem, or potential problem, is identified. Without morally courageous leaders, employees are not likely to point out wrongdoing or a lack of fairness for fear of retaliation. “Backlash is especially likely if powerful people in the organization benefit from the perpetuation of the very practices or systems ethical employees challenge” ([66], p. 117). Thus, a lack of moral courage from leaders will stifle moral courage from their subordinates. Without the courage to confront ethical problems and challenges to wellness within the organization, police leaders cannot begin to establish a culture of wellness and ethics.

Assuming that police leaders are morally courageous and have established the culture of wellness and ethics, it takes a certain kind of leadership to maintain those principles. The first area that requires exceptional police leadership relates to the emotional and spiritual distress that officers face from the moral risks of policing. It seems archaic, but some organizations continue to stigmatize officers who suffer from psychological problems even if those agencies employ mental health professionals. Those services, then, are more a function of risk management efforts than they are a reflection of a culture of wellness. Even police leaders who mean well when it comes to officer wellness are often a by-product of their upbringing in the profession and are underprepared to effectively address the job’s moral risks or their officers’ emotional and spiritual needs. With a record numbers of police suicides [67], it should be a top priority for all police leaders to find new ways for the organization, as a whole, to take better care of each other.

Organizations with a strong culture of wellness recognize that injuries are part of the game. Regarding the moral risks, police leaders appreciate the extent to which their officers are vulnerable to the effects of moral distress, moral injury, compassion fatigue, and numerous other reactions to their chronic exposure to the routine and traumatic aspects of the job. Some officers will “play” hurt, but some will require a trip to the injured list. Those who continue to work while experiencing distress should receive the help they need, including the availability of light duty assignments if necessary. Moreover, officers who are temporarily off-duty due to an injury (physical or psychological) should not feel abandoned by the department, which commonly occurs when injuries are stigmatized. Instead, police leaders should ensure that these officers continue to feel valued and connected. After recovery, these officers should be welcomed back as the asset that they are. In fact, organizations with a strong culture of wellness encourage self-referral when officers are feeling distressed and reinforce that help is available and that there is no stigma attached to reaching out for assistance. These organizations would rather have officers respond to their own early warning signs of distress than to have others raise concerns after the officer’s performance is impacted by that distress. For that to succeed, it requires a paradigm shift to the belief that injuries are not a sign of weakness, which is a core component of a strong culture of wellness.

The second area that requires exceptional police leadership relates to the moral risks that increase the likelihood of misconduct. Perhaps the most important step that police leaders can take in this regard is to curtail practices that contribute to moral disengagement [2]. Initiatives can be enacted to prevent each of the eight mechanisms of moral disengagement [10] from infecting police work. (A description of these initiatives is beyond the scope of the paper, but interested readers are encouraged to contact the authors.) For example, dehumanization (“stripping people of human qualities,” [10], p. 200) of any community member cannot be tolerated in any form. Preventing this requires police leaders to demonstrate the moral courage to challenge the status quo by enacting changes in the recruiting and hiring process, revamping academy and field training, and implementing new oversight procedures. Indications of dehumanization, even in its subtle forms, and the other mechanisms of moral disengagement should be swiftly confronted and responded to with appropriate disciplinary actions.

A significant tool that police leaders can use to discourage moral disengagement and other moral risks that lead to misconduct is increased community engagement. Morally courageous leaders demonstrate a commitment to improving the agency’s relationship with the community, which can occur with or without implementation of traditional community-oriented policing practices. This is not an easy step to take, because it requires cooperation from all interested parties. On one hand, these efforts are often met with resistance and cynicism by community leaders. On the other hand, officers (and police union officials) usually convey tremendous skepticism when their leaders make these conciliatory gestures and accuse their leaders of “playing politics.” This is where the morally courageous leader takes charge to show all stakeholders the bigger picture: Improved relations between the police and the community make life better for community members and are a way to improve officer wellness. Building these bridges is not a capitulation. Rather it is a way to innovatively maintain the organization’s culture of wellness and ethics, which cannot be successfully accomplished within the agency alone.

Finally, effective leaders have humility. They understand that one can never know what one does not yet know. Humility drives effective leaders to discover what is not known, to remain curious and open minded to innovation, and to continually seek new ways to improve the on-duty experiences and off-duty lives of their officers. There are many areas in which policing is being challenged to improve. Fundamentally, these areas involve officers’ fitness. However, policing cannot progress unless leaders become change agents who have the moral courage to bring about much needed improvements in police officer wellness, ethics, and resilience.

## 6. Conclusions

The objective of this article was to provide a broad overview of strategies to address the moral risks of policing. These risks compromise officers’ wellness and impact their operational readiness. There are no easy solutions. Piecemeal attempts to improve wellness or to prevent misconduct fall short for two reasons. The first is that the moral risks synergistically impact each other. When wellness is compromised because of moral distress, ethical exhaustion, or compassion fatigue, there is an increased likelihood of misconduct. Following misconduct, officers may experience emotional and/or spiritual distress, which then can lead to more misconduct, among other outcomes. The second reason is that improving officer wellness and preventing misconduct requires an integrated organizational response.

There are solutions available to police leaders who are committed to improving officer wellness and reducing misconduct. Some of these are easier to implement than others and some require the full support and contributions of additional stakeholders, including police union leaders. The first step, however, is to conceptualize the inseparable connection between officer wellness and ethics. With this viewpoint as the foundation, comprehensive innovations can be implemented in all facets of the organization. Specifically, recruiting and hiring, training, supervision, and promotional practices can be updated to fundamentally emphasize officer wellness and ethics. This requires police leaders to be willing to challenge the status quo. Nevertheless, these important changes ultimately will improve both officer safety and the relations between police organizations and members of the community.
